# Advancing from perception to reality: How to accelerate and achieve gender equity now

**DOI:** 10.1007/s40037-019-00545-4

**Published:** 2019-11-21

**Authors:** Tiffany I. Leung, Eileen Barrett, Tammy L. Lin, Darilyn V. Moyer

**Affiliations:** 1 Department of Internal Medicine, Maastricht University Medical Center +, Maastricht University, Maastricht, The Netherlands; 2 Care and Public Health Research Institute, Maastricht University, Maastricht, The Netherlands; 3 Division of Hospital Medicine, Department of Internal Medicine, University of New Mexico, Albuquerque, NM USA; 4 Med Mindset, San Diego, CA USA; 5 Voluntary Faculty, Department of Medicine, University of California, San Diego, Health Sciences, San Diego, CA USA; 6 American College of Physicians, Philadelphia, PA USA

We applaud Lukela et al. for contributing their institutional experience to the rapidly growing literature that quantifies the numerous and insidious ways that gender inequity manifests in medicine. The study found that women resident physicians perceived underrepresentation of women physician faculty to a greater extent than male resident physicians. Comparing perception to actual representation, they found that women residents noticed the gender disparity in leadership more often than did male residents [1].

Yet the findings of this single-institution survey study in Michigan were largely unsurprising. The data are increasingly clear that women physicians have less access to mentors, sponsors, and leadership positions [2, 3]. They are also less likely to be recommended as a discussant or panelist for national or international meetings, or serve on an editorial board or a national committee. Furthermore, women in academics are more likely than men to take on both internal and external service [4] or volunteer for ‘invisible work’ that is less likely to lead to promotions [5, 6]. Viewing the leadership pipeline, low inflow [7, 8] and high outflow of women faculty leaving, due to a lack of professional advancement or low salary [9], seem to be root causes for the symptom that Lukela et al. observed.

Such inequities are not unique to the United States. In a study that surveyed clinical faculty in Singapore, Qatar, and the United Arab Emirates, only one quarter of clinician educators were women, and they were more likely to be younger, single, and without children [10]. One review described women physicians’ work in Japan, Scandinavia, Russia, and Eastern Europe, purposively selecting these regions for their differences in cultural perspectives of gender roles, workforce policies, and environments, and their variety in their gender inequality indices [11]. Regardless of the proportion of female medical students or women physicians in early career, attainment of specialist or leadership positions in medicine remained low for similar reasons: structural barriers such as inflexible working hours, especially relating to work-family conflict, gender discrimination, or lack of role models [11].

Medicine across the globe is positioned to take decisive action now to advance gender equity in medicine. In particular, organizational leadership should design a *just, equitable, diverse,* and *inclusive *(JEDI) environment, as part of a larger effort towards organizational professionalism [12]. Driving gender equity forward is a necessary part of this effort because without transformational change, at the current rate of closure of the compensation gap by gender, achieving equity in this domain could take well over a century [13]. If this signals the rate of change towards gender equity in *all *domains, we are in dire need of big, bold steps forward to more rapidly achieve equity in career advancement and leadership.

Without bold steps forward, the cycle self-perpetuates: women physicians in faculty positions are less likely to advance, more likely to leave, and trainees will continue to find few women physician mentors, sponsors, and role models. How can we break this cycle? What seismic transformations can leaders implement to accelerate gender equity in medicine? To achieve gender equity, we offer the following recommendations for organizations and their leaders:

*Role model organizational professionalism and ethics*. Organizational professionalism envisions aspirations that, among other aims, includes organizational culture change to promote worker well-being [10]. The* Be Ethical Campaign* further frames this foundational necessity for gender diversity and inclusivity in healthcare institutions and organizations as a matter of organizational ethics [14]. Collecting data on structural inequities, including leadership positions, awards, and recognition, for example, and developing policies and practices to address identified inequities are high-priority tasks—which our organization, the American College of Physicians (ACP), is taking on. Few professional medical organizations have already led the way, and medical education organizations, such as the Accreditation Council for Graduate Medical Education, Liaison Committee on Medical Education, and the Association of American Medical Colleges (AAMC), could similarly role model this organizational standard. Moreover, harmonization of such efforts across global medical organizations would maximize the impact of advances towards gender equity in medicine everywhere.

*Champion the study of gender equity to dismantle barriers in promotion and tenure and engage in benchmarking with public reporting from other organizations.* Organizations’ leaders, including deans of diversity, inclusion, and equity, or even C‑suite executives, would show through action that they are prioritizing and valuing highly qualified women physicians. Deliberate design of an ‘escalator’ of career advancement towards leadership roles for women would support organizational efforts to utilize its best talents [15]. Extending the metaphor further, women physicians often step off the escalator due to socialized gender roles and expectations (e.g. to give birth and provide a dominant proportion of childcare), but find themselves at a later time willing but unable to step back onto the escalator. Even in countries where women physicians may be well-represented at the trainee and early career stages, their nonlinear route upwards is perceived and acted on as a barrier to career advancement, rather than valued for the diversity of experience it adds to their leadership qualifications that could further promote organizational professionalism.

*Address structural biases*. As healthcare organizations introspect about equity, identifying disparities in the physician pipeline, they can begin to redesign policies and practices that perpetuate structural biases. For example, implementing family-friendly policies can mitigate bias and discrimination. Formal programs, peer mentorship, or coaching can also help improve mentorship, sponsorship, and advancement overall [16]. Leadership training for physicians in all career stages could support the needed knowledge and skills towards policy development, such as those implemented by the AAMC [17], consortia of professional medical organizations, including our own [18], and others [19]. Such programs offer opportunities for retaining women physicians in the career pipeline and on the escalator towards leadership.

*Cross-pollinate different modes of thought towards the achievement of gender equity*. The Time’s Up initiative began in Hollywood to address workplace sexual harassment but quickly seeded the current Time’s Up Healthcare movement. Addressing sexual harassment through organizational policies and perhaps also training is necessary to mitigate its significant effects on women physicians’ confidence and their ability to advance in their careers [20]. In a different mode of thought, the quality improvement mindset, often applied to improve the quality of patient care, could also help the medical community achieve gender equity. Finally, all of these efforts described so far synergize with efforts to promote physician and trainee well-being, especially for women physicians who experience unique barriers and challenges in burnout [21]. Academic medical centres are uniquely positioned to address each of these challenges contemporaneously.
